# Trophoblast metabolic reprogramming triggers adverse pregnancy outcomes during DENV-2 infection in mice

**DOI:** 10.1128/jvi.00300-26

**Published:** 2026-08-21

**Authors:** Han Wang, Fei-Yang Xue, Shi-Qi He, Jin-Fan Yang, Hao Zhang, Jia-Ying Cao, Jia-Tong Chang, Chen Zhang, Jing An, Zi-Yang Sheng, Pei-Gang Wang

**Affiliations:** 1 Department of Microbiology, School of Basic Medical Sciences, Capital Medical University 12517, Beijing, China; 2 Laboratory for Clinical Medicine, Capital Medical University 12517, Beijing, China; 3 Department of Blood Transfusion, Peking University Third Hospital 66482, Beijing, China; The University of Texas Southwestern Medical Center, Dallas, Texas, USA

**Keywords:** adverse pregnancy outcome, dengue virus, syncytialization, metabolic reprogram

## Abstract

Adverse pregnancy outcomes associated with dengue virus (DENV) infection are a severe manifestation of dengue fever; however, we still poorly understand the underlying mechanisms. Trophoblast dysfunction in the placenta contributes centrally to various pregnancy complications. As the functional unit of the placenta, the syncytiotrophoblast (STB) requires continuous fusion of precursor trophoblasts and adequate energy supply. In this study, we established a model of adverse pregnancy by infecting *Ifnar*1^−/−^ pregnant mice with DENV-2. We did not detect viral replication within the placental trophoblast layer but observed substantial structural damage to the STB. RNA-seq analysis demonstrated that precursor trophoblasts exhibit disrupted glucose metabolism, and placental tissue upregulates pro-fusion genes—yet fails to complete syncytialization. Metabolic assays revealed reduced ATP levels and elevated lactate concentrations, indicating that an inadequate energy supply impedes trophoblast differentiation. Treatment with the glycolytic inhibitor 2-DG restored oxidative phosphorylation (OXPHOS) activity in the placenta and markedly improved pathological outcomes. Further mechanistic studies established that DENV infection induces oxidative stress, which activates hypoxia-inducible factor Hif1α, leading to subsequent upregulation of lactate dehydrogenase (LDHA) expression. We identify this cascade as a fundamental mechanism underlying metabolic reprogramming in trophoblasts. Our findings not only clarify the key pathological processes driving DENV-induced adverse pregnancy outcomes but also propose novel therapeutic strategies for clinical intervention.

**IMPORTANCE:**

Dengue virus infection during pregnancy is linked to adverse fetal outcomes, but the underlying mechanisms remain unclear. In *Ifnar*1^−/−^ pregnant mice, we show that fetal growth restriction is driven by disruption of the maternal environment that impairs placental function. We identify syncytiotrophoblast damage and defective trophoblast fusion despite compensatory upregulation of pro-fusion genes. Placental metabolic analysis reveals a shift from oxidative phosphorylation to glycolysis, accompanied by reduced ATP production and impaired syncytialization. Inhibition of glycolysis *in vivo* partially restores placental structure and improves fetal outcomes, supporting a key role for metabolic reprogramming. Mechanistically, ROS activates HIF-1α and upregulates its downstream target LDHA, thereby linking metabolic reprogramming to placental dysfunction and highlighting this pathway as a potential therapeutic target. These findings reveal a mechanism underlying DENV-associated pregnancy complications and highlight metabolic pathways as potential therapeutic targets.

## INTRODUCTION

Dengue virus (DENV) infection has increasingly been recognized as a threat to maternal-fetal health (1, 2), with epidemiological studies linking maternal dengue to preterm birth, low birth weight, fetal distress, and elevated perinatal morbidity (3–5). Despite these associations, the mechanisms by which DENV disrupts placental function remain poorly understood. Our previous work established a DENV-2 infection model in pregnant *Ifnar*1^−/−^ mice, in which timed pregnancies were generated and mice were inoculated with 10⁵ PFU of DENV-2 at E12.5, followed by analysis at E18.5. Using this model, we demonstrated that DENV-2 infection impairs placental blood flow through neutrophil-mediated vascular disruption (6), yet how trophoblast function is affected during this process has not been clarified.

The syncytiotrophoblast (STB) forms the primary maternal-fetal interface and relies on continuous fusion of cytotrophoblasts (CTBs), a process known as syncytialization (7). Proper syncytialization is essential for maintaining barrier integrity, nutrient exchange, and fetal growth (8). Disruption of STB formation has been implicated in multiple pregnancy complications that resemble those observed in DENV-infected pregnancies. During murine placental development, trophoblast fusion and the establishment of the labyrinth layer are rapidly initiated in early gestation and progressively attenuate as gestation advances (9, 10). Syncytiotrophoblast types I and II both derive from labyrinth trophoblast progenitors (LaTPs), which correspond to human CTBs (11). Notably, trophoblast progenitors remain dynamically regulated under stress or pathological conditions, exhibiting enhanced proliferative activity despite an overall reduction in cell number, supporting sustained and adaptable trophoblast renewal and fusion (12).

Trophoblast fusion is highly energy-dependent and requires a balance between oxidative phosphorylation (OXPHOS) and glycolysis (13, 14). CTBs commonly adopt aerobic glycolysis during proliferation but must transition to OXPHOS as they differentiate into STBs. Stressors that disrupt this metabolic switch, such as metabolic stress (15), oxidative stress (16), or mitochondrial dysfunction (17), can impair syncytialization, leading to structural and functional defects. Whether DENV infection perturbs trophoblast metabolic programming, thereby impairing syncytialization, remains unknown.

Therefore, in this study, we investigated how DENV-2 infection alters trophoblast differentiation and metabolic homeostasis, and whether metabolic reprogramming contributes to syncytiotrophoblast injury and adverse pregnancy outcomes.

## MATERIALS AND METHODS

### Viruses, cells, and animals

Dengue virus serotype 2 (DENV-2, strain Tr1751) was kindly provided by Dr. Kotaro Yasui (18, 19). After receipt, the virus was propagated exclusively in Aedes albopictus C6/36 cells and was never maintained in mammalian hosts or cell lines to minimize *in vitro* adaptive mutations. Low-passage aliquots were used for all experiments, and viral titers were routinely determined by plaque-forming assay using African green monkey kidney (Vero) cells to ensure experimental consistency. The human choriocarcinoma cell line BeWo, a well-characterized cell model of human placental trophoblasts, was cultured in Ham’s F12K media (Kaighn’s; Gibco) supplemented with 10% FBS. BeWo cells were cultured under standard conditions and used for experiments at approximately 70% confluence. To induce syncytialization, cells were treated with 50 μM forskolin (FSK) for 48 h. Oxidative stress was induced by treatment with 100 μM hydrogen peroxide (H₂O₂) for 6 h. To inhibit HIF-1α signaling, cells were treated with the pharmacological inhibitor YC-1 at concentrations of 0, 5, 10, and 20 μM. YC-1 was added 1 h prior to H₂O₂ treatment and maintained throughout the duration of the experiment. All cell lines were maintained at 37°C in a 5% CO₂ atmosphere.

Type I interferon receptor knockout (*Ifnar*1^−/−^) mice were purchased from the Institute of Zoology, Chinese Academy of Sciences. To establish the pregnancy model, 8- to 10-week-old female *Ifnar*1^−/−^ mice were housed with male *Ifnar*1^−/−^ mice at a 2:1 ratio. The presence of a vaginal plug the following morning was designated as embryonic day 0.5 (E0.5). On E12.5, pregnant mice were inoculated via the footpad with 10⁵ PFU of DENV-2 to establish infection; control mice received an equal volume (100 μL) of sterile PBS. Mice were euthanized at E15.5 and E18.5, and maternal blood and placentas were collected for viral load quantification, histological analysis, single-cell RNA sequencing, and metabolomics. For the glycolysis inhibition assay, pregnant *Ifnar*1^−/−^ mice from both DENV-2-infected and uninfected groups were randomly selected. Starting from E12.5 until E18.5, mice received a daily intraperitoneal injection of 500 mg/kg 2-deoxy-D-glucose (2-DG, Macklin, Cat# D807272), which was dissolved in saline to the target concentration. The selected dose was based on previously published *in vivo* studies in which 2-DG was used to modulate glucose metabolism within a tolerable range (20). For the inhibition test, *Ifnar*1^−/−^ pregnant mice were injected with vehicle or GSH (8 mg, dissolved in PBS) daily from E12.5 to E17.5 after infected with 10^5^ PFU DENV-2, as previously described in our prior study (21). GSH was purchased from Solarbio. For the *in vivo* rescue experiment, the HIF-1α-specific inhibitor PX-478 (25 mg/kg) was administered daily via intraperitoneal injection starting from E12.5 until E18.5. PX-478 was purchased from MCE (HY-10231).

Fetal and placental samples were collected from different dams, and measurements were averaged per dam to avoid pseudoreplication. Each group had 4–5 pregnant dams. All pregnant mice successfully mated and confirmed by the presence of a vaginal plug at E0.5 were included. Animals were excluded only if they died before the planned endpoint or if pregnancy was not confirmed; however, neither of these events occurred in this study. All inclusion and exclusion criteria were pre-established prior to the experiment. For all analyses, the experimental unit was a single pregnant dam. To minimize confounders, animals were housed under identical environmental conditions (temperature, humidity, light-dark cycle, and access to food/water). Cage location on the rack was rotated weekly to avoid positional bias. Treatments and sample collection were performed in alternating order across groups to prevent batch or temporal effects.

### Hematoxylin and eosin staining

Placental tissues from DENV-2-infected and uninfected *Ifnar*1^−/−^ mice were paraffin-embedded and sectioned at a thickness of 5 μm. Tissue sections were stained with hematoxylin for nuclear visualization, differentiated in 0.5% acid-alcohol solution, and counterstained with eosin for cytoplasmic staining.

### Immunofluorescence staining

To visualize trophoblast morphology and microvasculature, placental sections were incubated with antibodies against MCT4 (Santa Cruz, sc-376140), MCT1 (Thermo Fisher, PA5-72957), CD34 (Abcam, ab81289), and EpCAM (Thermo Fisher, MA5-12436). Cell proliferation was assessed using an anti-Ki67 antibody (Abcam, ab15580). Metabolic changes were evaluated with anti-HIF-1α (Huaxingbio, HX20196) and anti-LDHA (Thermo Fisher, PA5-27406) antibodies. Viral antigen distribution was detected using a lab-prepared mouse polyclonal anti-flavivirus antibody. CD71 (Santa Cruz, sc-65882) was used as a marker for BeWo-derived syncytiotrophoblasts. Oxidative stress was visualized using the fluorescent probe dihydroethidium (DHE, Beyotime, S0064S). All primary antibody incubations were performed overnight at 4°C. Subsequently, sections were incubated with a donkey anti-rabbit secondary antibody (1:1,000, Life Technologies, A21207), and nuclei were counterstained with DAPI-containing mounting medium (ZSGB-BIO, ZLI-9557).

### Quantitative real-time PCR

Total RNA was extracted using TRIzol reagent (TransGen Biotech, ET101-01-V2). RNA levels were quantified using the Quant One Step RT-PCR Kit (SYBR Green, TIANGEN, FP313-01) on a QuantStudio 5 Real-Time PCR System (Applied Biosystems, USA). Primer sequences for DENV-2, *Gapdh, Syna, Synb, Gcm*1, *ERVW*-1, and *ERVFRD*-1 are listed in Table S1.

### Single-cell RNA sequencing (scRNA-seq)

Placental tissues were dissociated into single-cell suspensions with a viability of ≥85% and a concentration of 700–1,200 cells/μL. cDNA amplification, library construction, and sequencing were performed following the manufacturer’s standard protocols (LC-Bio Technology Co., China). The uniform manifold approximation and projection (UMAP) method was used for data visualization. Cell clusters were identified based on similarity and annotated using marker gene expression. Gene set enrichment analysis (GSEA) was subsequently performed on specific cell subsets to investigate their functional characteristics.

### Targeted metabolomics analysis

Metabolomic analysis was conducted using ultra-performance liquid chromatography (1290 Infinity LC, Agilent Technologies) coupled with quadrupole time-of-flight mass spectrometry (AB Sciex TripleTOF 6600) at Shanghai Majorbio Biomedical Technology Co., Ltd. Data analysis was performed on the Majorbio cloud platform.

### Enzyme-linked immunosorbent assay

Concentrations of VEGF, PGF, and β-CG in *Ifnar*1^−/−^ mouse placentas were quantified using commercial Mouse VEGF (Sangon Biotech, D721156), Mouse PGF (Sangon Biotech, D721358), and Mouse β-CG (MEIMIAN, MM-0581M2) ELISA kits, respectively. Concentration of β-hCG in BeWo cells was quantified using a commercial Human β-hCG ELISA kit (JL117260). Absorbance was measured at 450 nm using a Multiskan Spectrum microplate reader (Thermo Fisher Scientific, USA).

### Detection of metabolic products and key enzymes

Lactate and ATP levels in placental homogenates were quantified using Lactate Assay Kit (njjcbio, A019-2-1) and ATP Assay Kit (njjcbio, A095-1-1), respectively. Activities of hexokinase (HK), pyruvate kinase (PK), and lactate dehydrogenase (LDH) in placental tissues were measured using specific activity assay kits (Solarbio). Activities of mitochondrial electron transport chain complexes I to V were determined using corresponding activity assay kits (adsbio). Absorbance was measured at specified wavelengths (530 nm or 550 nm) using a Multiskan Spectrum microplate reader (Thermo Fisher Scientific, USA).

### Quantification and statistical analysis

Pregnant dams were randomly assigned to experimental groups after confirmation of pregnancy (E0.5). Randomization was performed using a computer-generated randomization list (Excel RAND function). Allocation was conducted by an investigator who was not involved in outcome assessment.

For hematoxylin-eosin (H&E)-stained sections, the labyrinth zone area and vascular area were quantified using ImageJ software. For IF staining, fluorescence intensity was measured using ImageJ, while the counting of Ki67, EpCAM, HIF-1α, and LDHA positive cells was performed manually by at least two independent investigators.

All experiments were repeated at least three times. Data are presented as mean ± standard deviation. Data were first assessed for normality using the Shapiro-Wilk test and for homogeneity of variance using Levene’s test. For data sets meeting these assumptions, parametric tests were applied, including Student’s *t*-test for two-group comparisons and one-way ANOVA for multiple-group comparisons. For data violating normality or variance homogeneity, non-parametric tests such as the Mann-Whitney *U* test were used. Statistical analyses were performed using GraphPad Prism 9.0. The normality of data distribution was tested first; non-parametric tests were applied if the data were not normally distributed. A *P*-value of less than 0.05 was considered statistically significant.

## RESULTS

### DENV-2 does not directly infect trophoblasts but induces structural injury

**Fig 1:**
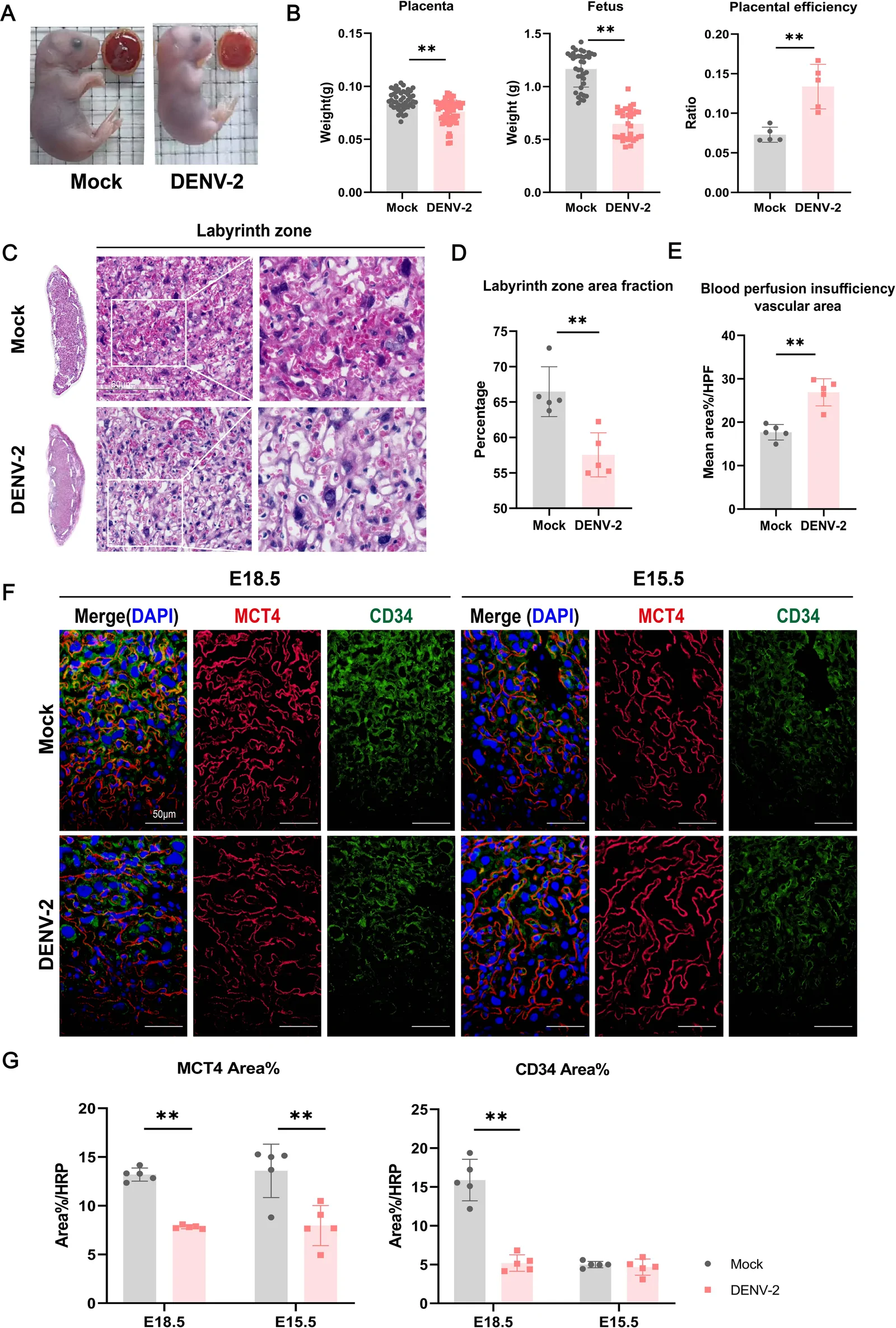
DENV-2 infection induces trophoblast injury in placentas of *Ifnar*1^−/−^ pregnant mice. At embryonic day (E)12.5, *Ifnar*1^−/−^ pregnant mice were infected by footpad injection with 10^5^ PFU of DENV-2; mock group received PBS. (**A**) Gross morphology of fetuses and placentas at E18.5. Both fetuses and placentas from DENV-2-infected dams appeared smaller than those from the mock group. (**B**) Placental weight, fetal weight, and placental coefficient at E18.5. *n* = 46–62 from 5 pregnant mice per group. (**C**) H&E staining of placentas from *Ifnar*1^−/−^ dams at E18.5. Left: longitudinal section of whole placenta, with the labyrinth zone outlined by a white dashed line. Scale bar, 3 mm. Right: representative pathology of the labyrinth. Scale bar, 60 μm. (**D**) Quantification of the labyrinth-to-placenta area ratio. (**E**) Quantification of poorly perfused vascular areas at high magnification. *n* = 5 per group. (**F**) Immunofluorescence staining of syncytiotrophoblasts and fetal vessels at E15.5 and E18.5. SynT-II was labeled by anti-MCT4 (red), fetal vessels by anti-CD34 (green), and nuclei by DAPI (blue). Scale bar, 50 μm. Quantification of SynT-II and fetal vessel area fractions. (**G**) Quantification of SynT-II and fetal vessel area fractions at E18.5 and E15.5. *N* = 5 per group. Data are presented as mean ± SD. Statistical analysis was performed using a two-tailed unpaired Student’s *t*-test. *P*-values: **<0.01.

To model DENV-2 infection during late pregnancy and evaluate its consequences for fetal outcomes, pregnant *Ifnar*1^−/−^ mice were challenged with 10^5^ PFU DENV-2 at embryonic day (E)12.5; the mock group received PBS by the same route. Maternal blood, placentas, and fetal tissues were collected at E15.5 (3 dpi) and E18.5 (6 dpi) (Fig. S1A), and maternal body weight was monitored throughout pregnancy. Pregnant dams gained weight more slowly than those in the mock group after DENV-2 infection (Fig. S1B). Compared with the mock group, the DENV-2 infected group had smaller and paler placentas and visibly growth-restricted fetuses in gross examination at E18.5 (Fig. 1A). Both placental and fetal weights were significantly reduced, while the placental coefficient—a predictor of fetal growth outcomes—was elevated (Fig. 1B). Additionally, fetal crown-to-rump length was significantly reduced in the DENV-2 infected group, indicating impaired fetal developmental growth (Fig. S1C). Collectively, these findings demonstrated that DENV-2 infection in *Ifnar*1^−/−^ mice could lead to intrauterine growth restriction (IUGR).

As the placenta serves as a critical interface between mother and fetus, we examined histopathological changes following DENV-2 infection at E18.5. H&E staining revealed that DENV-2-infected placentas decreased thickness of the labyrinth zone (Fig. 1C), and quantitative analysis confirmed the reduction of the labyrinth-to-placenta area ratio (Fig. 1D). In addition, DENV-2-infected placentas exhibited inflammatory cell infiltration and impaired vascular perfusion, as evidenced by an increased proportion of empty vessels (Fig. 1E). Since the labyrinth zone forms the main component of the fetomaternal barrier—comprising trophoblast layers and fetal vasculature—we next investigated whether DENV-2 infection disrupts the integrity of these cellular compartments. Syncytiotrophoblast type II is located adjacent to fetal microvessels and plays a key role in maintaining barrier integrity and maternofetal exchange (22). Therefore, we used SynT-II as a representative indicator of trophoblast barrier disruption. Immunofluorescence for syncytiotrophoblast type II (MCT4) and microvessels (CD34) was damaged compared with the mock group at E18.5 (Fig. 1F and G). Importantly, MCT4 area revealed a significant loss in the mock group at an earlier point (E15.5), while CD34 positive vessel density remained comparable to the mock group (Fig. 1F through H), indicating that syncytiotrophoblast injury precedes over microvascular collapse. Vascular endothelial growth factor (VEGF) and placental growth factor (PlGF), secreted by syncytiotrophoblasts to repair vessels (23), were increased after DENV-2 infection (Fig. S1D), suggesting compensatory upregulation in response to vascular disruption.

Taken together, these data are consistent with a model in which Syncytiotrophoblast type II disruption compromises the physical support for fetal microvessels, contributing to subsequent vascular collapse and the fetomaternal barrier impairment that may lead to fetal growth restriction.

### Trophoblast impairment after DENV-2 infection results from abnormal syncytialization

**Fig 2:**
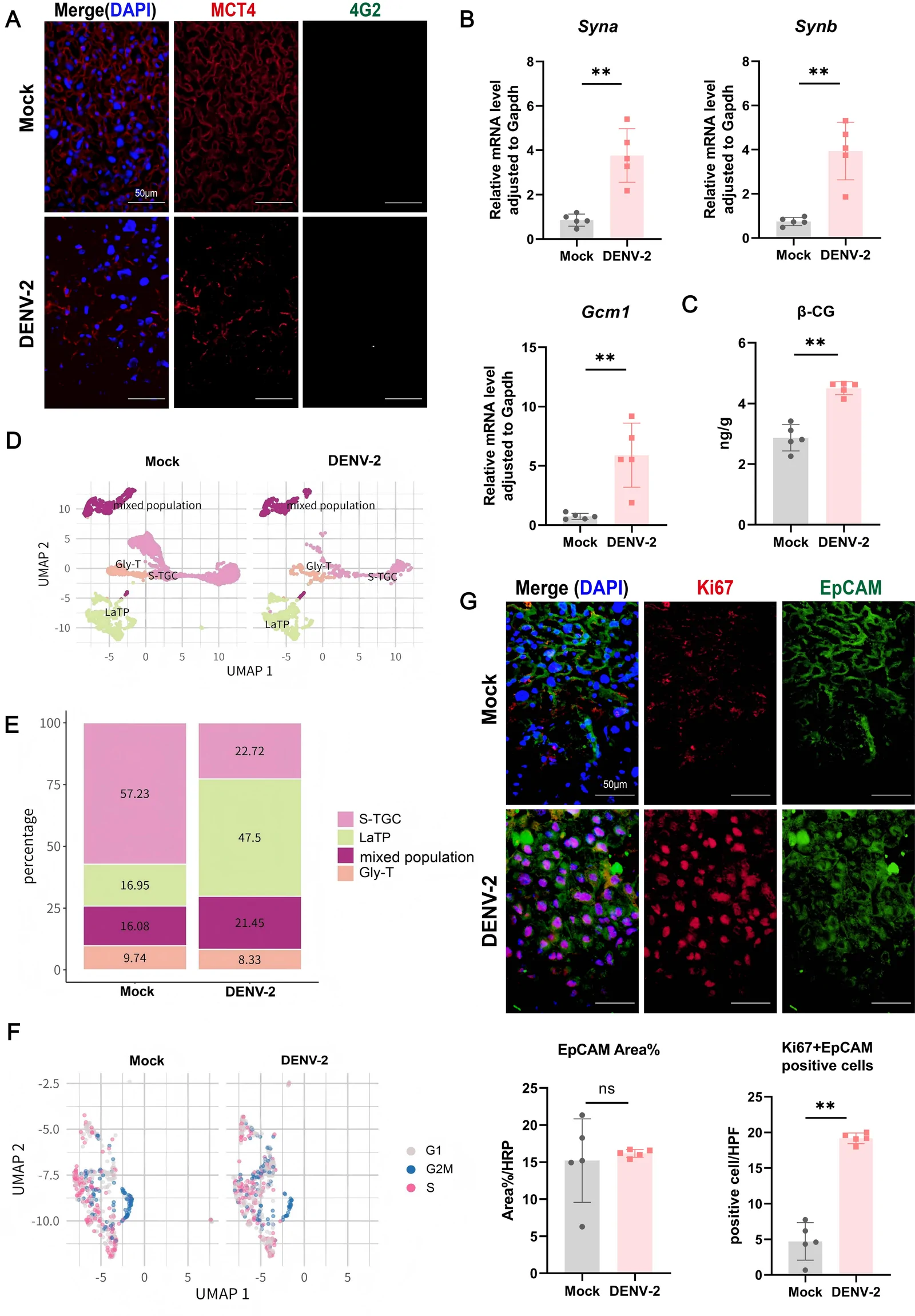
DENV-2 infection disrupts trophoblast fusion in *Ifnar*1^−/−^ placentas. At embryonic day (E)12.5, *Ifnar*1^−/−^ pregnant mice were infected by footpad injection with 10^5^ PFU of DENV-2; mock group received PBS. Placentas were collected at E18.5 for scRNA-seq analysis. (**A**) Immunofluorescence staining of syncytiotrophoblasts. SynT-II was labeled with anti-MCT4 (red), flavivirus E protein with anti-4G2 (green), and nuclei with DAPI (blue). Scale bar, 50 μm. (**B**) RT-qPCR analysis of syncytial fusogenic genes (*Syna, Synb, Gcm1*). (**C**) ELISA measurement of β-CG levels. (**D**) UMAP visualization of annotated trophoblast subpopulations. (**E**) Proportional distribution of trophoblast subpopulations. (**F**) Cell-cycle state of LaTPs (G1, S, G2/M) shown by UMAP in mock and DENV-2-infected groups. (**G**) Immunofluorescence staining of LaTP proliferation. Proliferating cells were labeled with anti-Ki67 (red), LaTPs with anti-EpCAM (green), and nuclei with DAPI (blue). Scale bar, 50 μm. Quantification of EpCAM^+^ cells and Ki67^+^ EpCAM^+^ cells. *N* = 5 per group. Data are presented as mean ± SD. Statistical analysis was performed using a two-tailed unpaired Student’s *t*-test. *P*-values: **<0.01. ns, not significant.

To investigate the underlying cause of syncytiotrophoblast injury, we first assessed whether differences in systemic and placental viral burden could account for the observed pathology. Quantitative analysis revealed comparable levels of DENV-2 RNA in maternal blood and placental tissues (Fig. S2A), indicating efficient systemic infection and effective viral exposure at the maternal-fetal interface. We next examined whether syncytiotrophoblast damage was associated with direct viral infection. Immunofluorescence staining using the pan-flavivirus antibody 4G2 and MCT4 detected little to no viral antigen within syncytiotrophoblasts (Fig. 2A), suggesting that productive infection of these cells is minimal and unlikely to be the primary driver of cellular injury.

To further characterize viral distribution within the placenta, immunofluorescence analysis revealed marked spatial heterogeneity, with limited viral signal in the labyrinth zone but more pronounced accumulation in the decidual region (Fig. S2C), indicating compartmentalized viral distribution at the maternal-fetal interface. Consistently, viral transcripts were predominantly enriched in placental macrophages, whereas trophoblast subsets exhibited minimal viral reads (Fig. S2B), identifying trophoblasts as non-permissive targets for active DENV-2 replication. Collectively, these findings suggest that syncytiotrophoblast injury is unlikely to result from direct viral replication but is more plausibly mediated by indirect mechanisms shaped by the placental microenvironment.

To further characterize trophoblast alterations in the placenta, placentas collected from *Ifnar*1^−/−^ mice were subjected to single-cell RNA sequencing (scRNA-seq). Trophoblastic cells in the placentas, identified by *Krt8* expression (24, 25), were reduced in DENV-2-infected group (Fig. S2D). To explore the underlying cause of trophoblastic cells reduction, common cell death processes were examined. The gene expression analysis revealed no marked changes in key regulators of apoptosis (*Caspase 2*, *3,* and *6*), ferroptosis (*Gpx4*, *Got1,* and *Acsl4*), or autophagy (*Atg12*, *Atg16l1,* and *Atg16l2*) upon DENV-2 infection (Fig. S2E), suggesting that trophoblast reduction was not associated with significant cell death.

After excluding direct viral infection and cell death as major contributors, defects in trophoblast proliferation or differentiation were next considered potential causes. Fusogenic genes (*Syna, Synb, Gcm1*) (22, 26) (Fig. 2B) and the pro-fusion hormone chorionic gonadotropin beta-subunit (β-CG) (27, 28) (Fig. 2C) exhibited compensatory upregulation in DENV-2-infected group. Nevertheless, this compensatory response failed to restore the syncytial layer. These findings suggest that the terminal differentiation and fusion of labyrinth trophoblast progenitors into mature syncytiotrophoblasts are impaired. Collectively, these results indicate that the fusion process is obstructed although placental cells exhibit a pro-syncytialization trend.

To further define trophoblast lineage alterations, trophoblast cells were clustered and annotated according to established surface markers (9) (Fig. S2F). This revealed four clusters including labyrinth progenitor trophoblasts (LaTP), sinusoidal trophoblast giant cells (S-TGC), glycogen trophoblasts (Gly-T), and a mixed population largely consisting of spongiotrophoblast cells (Fig. 2D). Quantification of trophoblast clusters as a percentage of total cells showed that LaTP was the only subset displaying a significant increase after DENV-2 infection (Fig. 2E). Cell cycle analysis showed that the expanded LaTP population in the DENV-2-infected group was enriched in cells in the G2/M and S phases (Fig. 2F), suggesting increased proliferation of LaTPs. To validate this proliferative activity, co-immunostaining was further performed using proliferation marker Ki67 and the LaTP surface marker EpCAM. The number of Ki67^+^ EpCAM^+^ cells significantly increased in the placentas of the DENV-2-infected group (Fig. 2G), confirming aberrant LaTPs proliferation.

Collectively, these results show that abnormal proliferation of LaTPs occurs in *Ifnar*1^−/−^ placentas after DENV-2 infection, while their differentiation appears compromised, which may contribute to defective syncytiotrophoblast development.

### Altered trophoblast respiratory metabolism underlies defective syncytialization following DENV-2 infection

**Fig 3:**
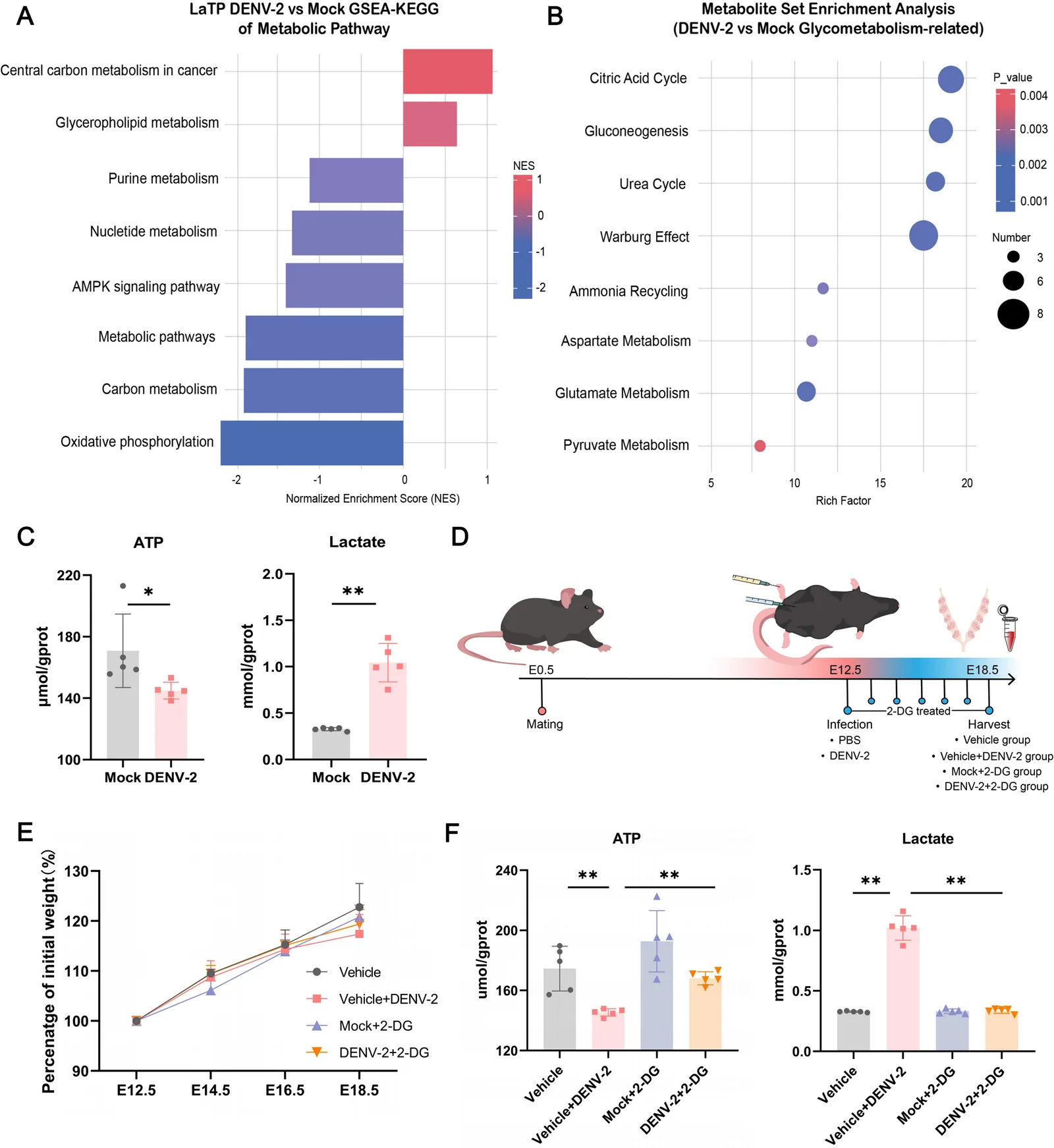
DENV-2 infection alters placental metabolism in pregnant *Ifnar*1^−/−^ mice. At embryonic day (E)12.5, *Ifnar*1^−/−^ pregnant mice were infected by footpad injection with 10^5^ PFU of DENV-2; mock group received PBS. Besides, at embryonic day (E)12.5, vehicle group received footpad injection of PBS and intraperitoneal injection of PBS, vehicle + DENV-2 group received footpad injection of 10^5^ PFU DENV-2 and intraperitoneal injection of PBS, mock + 2-DG group received footpad injection of PBS and intraperitoneal injection of 2-DG, DENV-2 + 2-DG group received footpad injection of 10^5^ PFU DENV-2 and intraperitoneal injection of 2-DG. (**A**) GSEA-KEGG analysis of differentially expressed metabolic genes in LaTP from DENV-2-infected vs mock groups. (**B**) Bubble plot of metabolite set enrichment analysis (MSEA) highlighting differences in gluconeogenesis-related metabolites between DENV-2-infected and mock groups in placental metabolomics of *Ifnar*1^−/−^ pregnant mice. (**C**) ATP and lactate levels in placental tissue from *Ifnar*1^−/−^ mice. (**D**) Experimental design: pregnant *Ifnar*1^−/−^ mice were injected via the footpad with 10^5^ PFU DENV-2 or PBS on embryonic day 12.5 (E12.5). From the day of infection until E18.5, mice received daily intraperitoneal injections of 2-DG (500 mg/kg). Blood and placentas were collected at E18.5 for downstream analyses. (**E**) Maternal body-weight trajectories following 2-DG treatment. (**F**) Placental ATP and lactate levels in *Ifnar*1^−/−^ mice after 2-DG treatment. *N* = 5 per group. Data are presented as mean ± SD. Statistical significance was determined by a two-tailed unpaired Student’s *t*-test in panel C and by one-way ANOVA followed by Tukey’s multiple comparisons test for comparisons among multiple groups in panels E and F. *P*-values: *<0.05, **<0.01.

To investigate the basis of the impaired fusion, LaTP cells were isolated by scRNA-seq. GSEA-KEGG analysis following DENV-2 infection revealed that upregulated genes were predominantly enriched in pathways related to enhanced inflammation, while downregulated genes were associated with metabolic and processes related to protein homeostasis and mitochondrial function (Fig. S3A). Notably, the general metabolic pathway was the most significantly downregulated, as indicated by the lowest normalized enrichment score (NES), highlighting a prominent suppression of metabolic activity in LaTPs after DENV-2 infection. To further dissect which aspect of metabolism was most affected, analysis was performed on KEGG pathways related to amino acid, lipid, and glucose metabolism. Among these, oxidative phosphorylation (OXPHOS) was the most significantly downregulated, pointing to a prominent impairment of glucose metabolism (Fig. 3A).

To further characterize this metabolic alteration at the tissue level, we performed placental metabolomics analysis. Principal component analysis (PCA) revealed a clear separation between DENV-2-infected and mock samples, indicating a distinct metabolic signature (Fig. S3B). Metabolite Set Enrichment Analysis (MSEA) comparing DENV-2-infected and mock groups revealed that the Warburg effect was among the most significantly upregulated pathways (Fig. 3B). These metabolomic findings complemented the single-cell data and prompted further transcriptional analysis of genes regulating OXPHOS and glycolysis.

We next performed an in-depth analysis of genes involved in the OXPHOS pathway. Compared with the mock group, the majority of upstream regulators of oxidative phosphorylation (e.g., *Ppargc1a*, *Tfam*, *Mfn2*) (29) were downregulated after DENV-2 infection, whereas upstream regulatory genes of Warburg (e.g., *Hif1α*, *Myc*, *Pdk1*) (30) were upregulated (Fig. S3C). To further validate these findings at the tissue level, the levels of ATP (the product of OXPHOS) and lactate (the product of the Warburg effect) were measured. ATP levels were decreased, and lactate levels were increased in the placentas of the DENV-2-infected group, confirming a metabolic shift from OXPHOS to Warburg effect within the placental cells of *Ifnar*1^−/−^ pregnant mice following DENV-2 infection (Fig. 3C).

To determine the contribution of altered respiratory metabolism to adverse pregnancy outcomes, we employed 2-deoxy-D-glucose (2-DG), a glucose analog that competitively inhibits glycolysis *in vivo* (31, 32). By interfering with glucose utilization, 2-DG is widely used to probe the functional role of glycolytic metabolism in physiological and pathological contexts. Based on the metabolic findings, 2-DG was administered daily via intraperitoneal injection to both DENV-2-infected and mock groups in the pregnant *Ifnar*1^−/−^ mice from the day of infection until E18.5 (Fig. 3D). Following 2-DG treatment, the body weight of DENV-2-infected pregnant mice trended toward normalization (Fig. 3E). To verify the *in vivo* efficacy of 2-DG, lactate and ATP levels were measured in the placentas (Fig. 3F). 2-DG treatment significantly reduced lactate content and partially increased ATP levels in DENV-2-infected placentas, whereas no changes were observed in the mock group. These results demonstrate that 2-DG effectively suppresses glycolysis and restores OXPHOS function *in vivo*.

**Fig 4:**
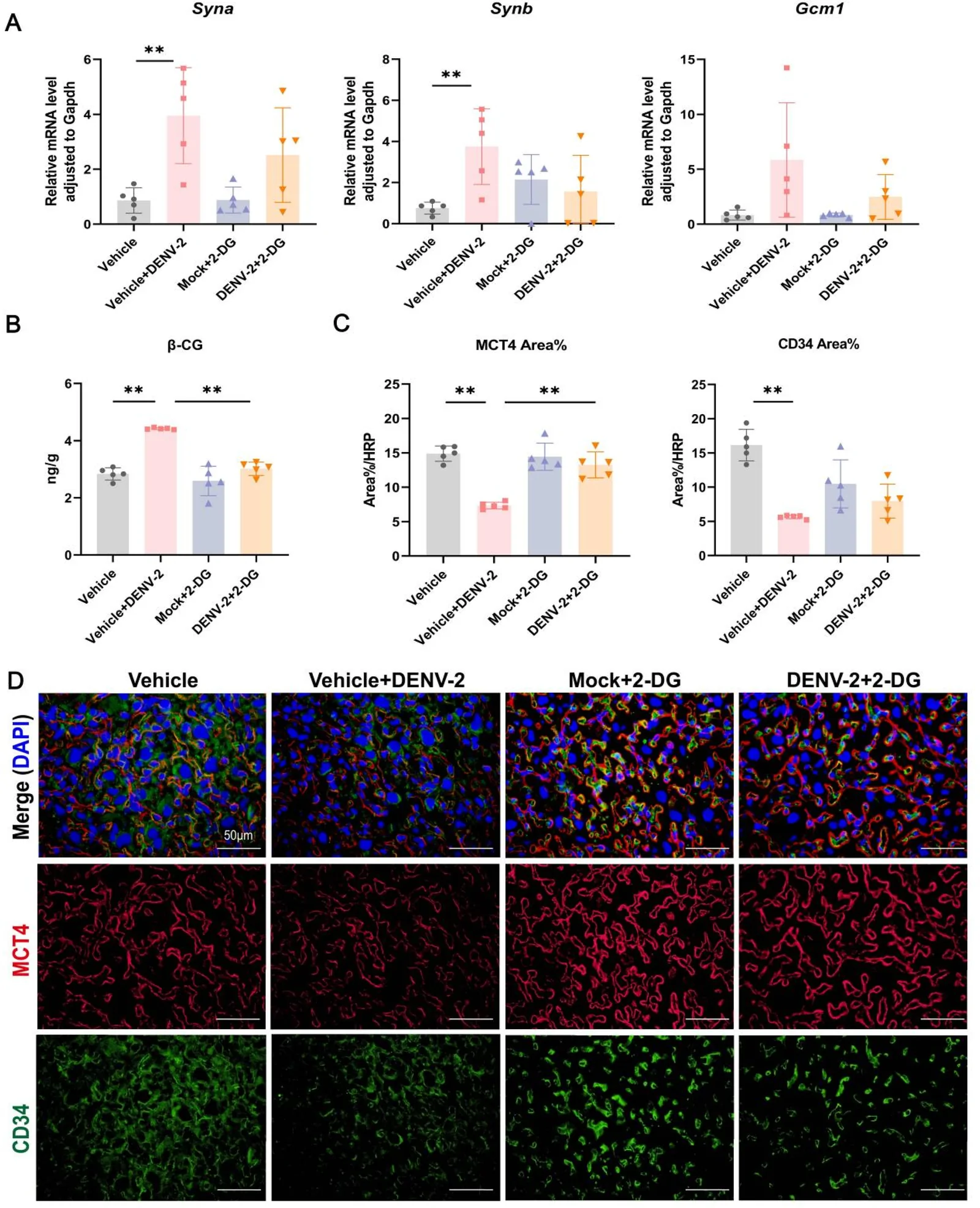
2-DG treatment mitigates syncytiotrophoblast injury in DENV-2-infected *Ifnar*1^−/−^ mice (**A**) RT-qPCR analysis of fusogenic genes (*Syna, Synb, Gcm1*) in placentas from 2-DG-treated *Ifnar*1^−/−^ mice. (**B**) ELISA quantification of β-CG levels in placentas after 2-DG treatment. (**C and D**) Immunofluorescence analysis of syncytiotrophoblasts and microvasculature after 2-DG treatment. SynT-II marked with anti-MCT4 (red), fetal microvessels marked with anti-CD34 (green), nuclei counterstained with DAPI (blue). Scale bar, 50 μm. Percent area of SynT-II and microvessels was quantified. *N* = 5 per group. Data are presented as mean ± SD. Statistical significance was determined by one-way ANOVA followed by Tukey’s multiple comparisons test. *P*-values: **<0.01.

We next examined whether inhibition of glycolysis by 2-DG could partially modulate trophoblast differentiation. RT-qPCR analysis revealed that *Syna*, *Synb*, and *Gcm1* levels were modestly reduced after 2-DG treatment in DENV-2-infected placentas (Fig. 4A). β-CG levels were restored to near-normal in DENV-2-infected mice following 2-DG treatment (Fig. 4B). Immunofluorescence for MCT4 and CD34 further demonstrated structural restoration of the syncytiotrophoblast layer and showed altered vascular organization (Fig. 4C and D). ELISA analysis showed that VEGF and PIGF were comparable between 2-DG-treated DENV-2-infected and vehicle control groups (Fig. S3D).

**Fig 5:**
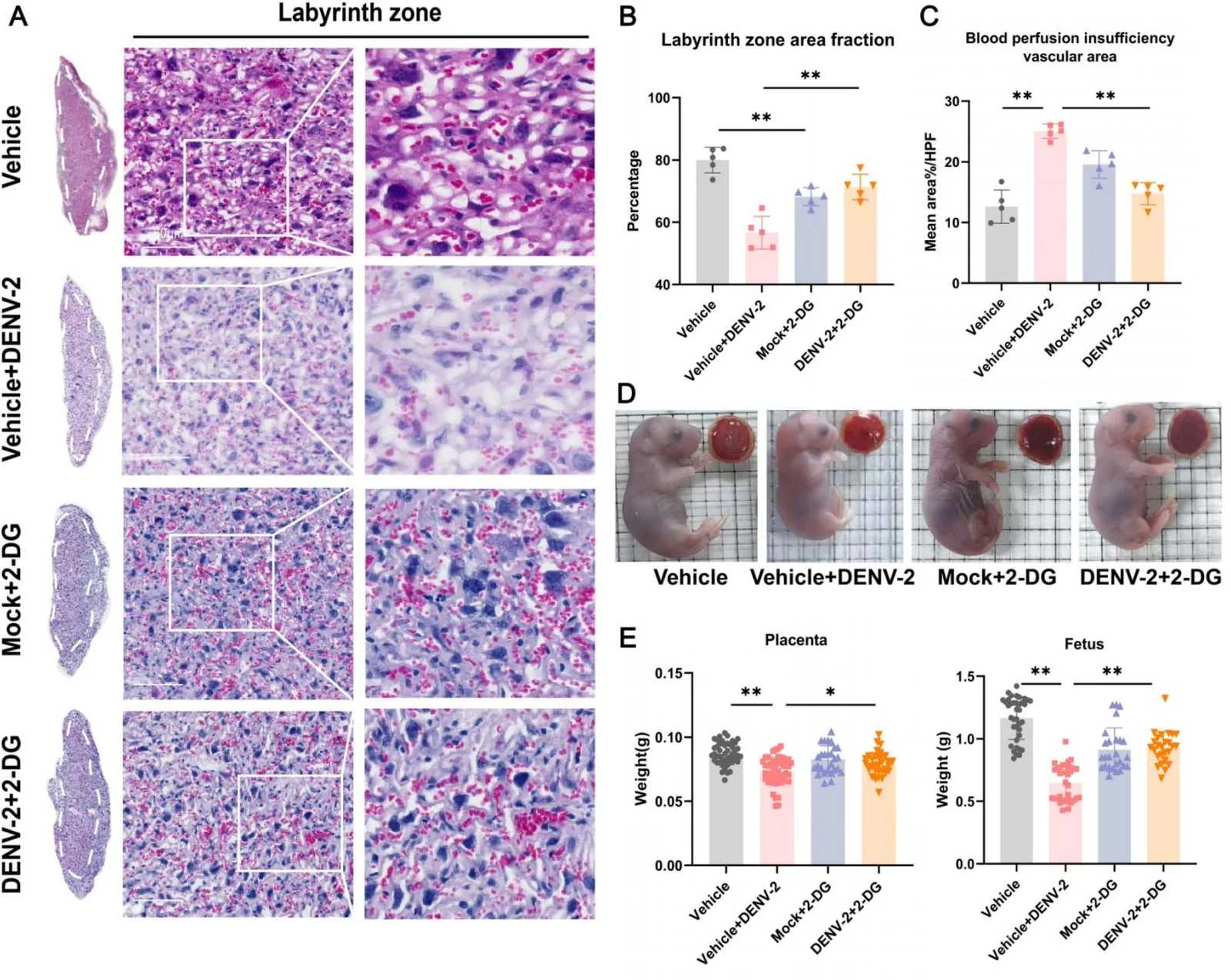
2-DG treatment mitigates placental and fetal injury in DENV-2-infected *Ifnar*1^−/−^ mice (**A**) H&E staining of placentas from 2-DG-treated *Ifnar*1^−/−^ mice. Left, longitudinal section of the whole placenta, with the labyrinth region outlined by dashed lines. Scale bar, 3 mm. Right, representative pathology of the labyrinth region. Scale bar, 60 μm. (**B**) Quantification of labyrinth area proportion in placentas after 2-DG treatment. (**C**) Quantification of poorly perfused vascular areas in placentas under high magnification after 2-DG treatment. (**D**) Gross morphology of fetuses and placentas from *Ifnar*1^−/−^ mice after 2-DG treatment. (**E**) Placental weight and fetal weight of *Ifnar*1^−/−^ mice at E18.5 after 2-DG treatment. *n* = 27–47 from 4–5 pregnant mice per group. *N* = 5 per group. Data are presented as mean ± SD. Statistical significance was determined by one-way ANOVA followed by Tukey’s multiple comparisons test. *P*-values: *<0.05, **<0.01.

H&E results showed an increase in the thickness of the labyrinth zone in 2-DG-treated, DENV-2-infected placentas compared with vehicle-treated counterparts (Fig. 5A and B). In addition, 2-DG treatment showed partial changes in perfusion (Fig. 4C). In contrast, 2-DG administration in mock mice led to a slight increase in avascular areas compared with vehicle controls. To determine whether 2-DG treatment could ameliorate DENV-2-induced IUGR, the gross morphology of placentas and fetuses was examined. Fetuses and placentas in 2-DG-treated, DENV-2-infected *Ifnar*1^−/−^ dams were larger than the vehicle and DENV-2-infected group (Fig. 5D), supported by increased fetal and placental weights (Fig. 5E).

Collectively, these results indicate that inhibition of glycolysis by 2-DG modulates metabolic status and is associated with partial changes in trophoblast function and placental structure. Together with the metabolomic and single-cell transcriptomic findings, these data support a role for metabolic reprogramming in DENV-2-associated placental dysfunction and adverse pregnancy outcomes.

### Enhanced glycolytic flux and ROS-HIF-1α-LDHA axis drive metabolic reprogramming in DENV-2-infected placentas

**Fig 6:**
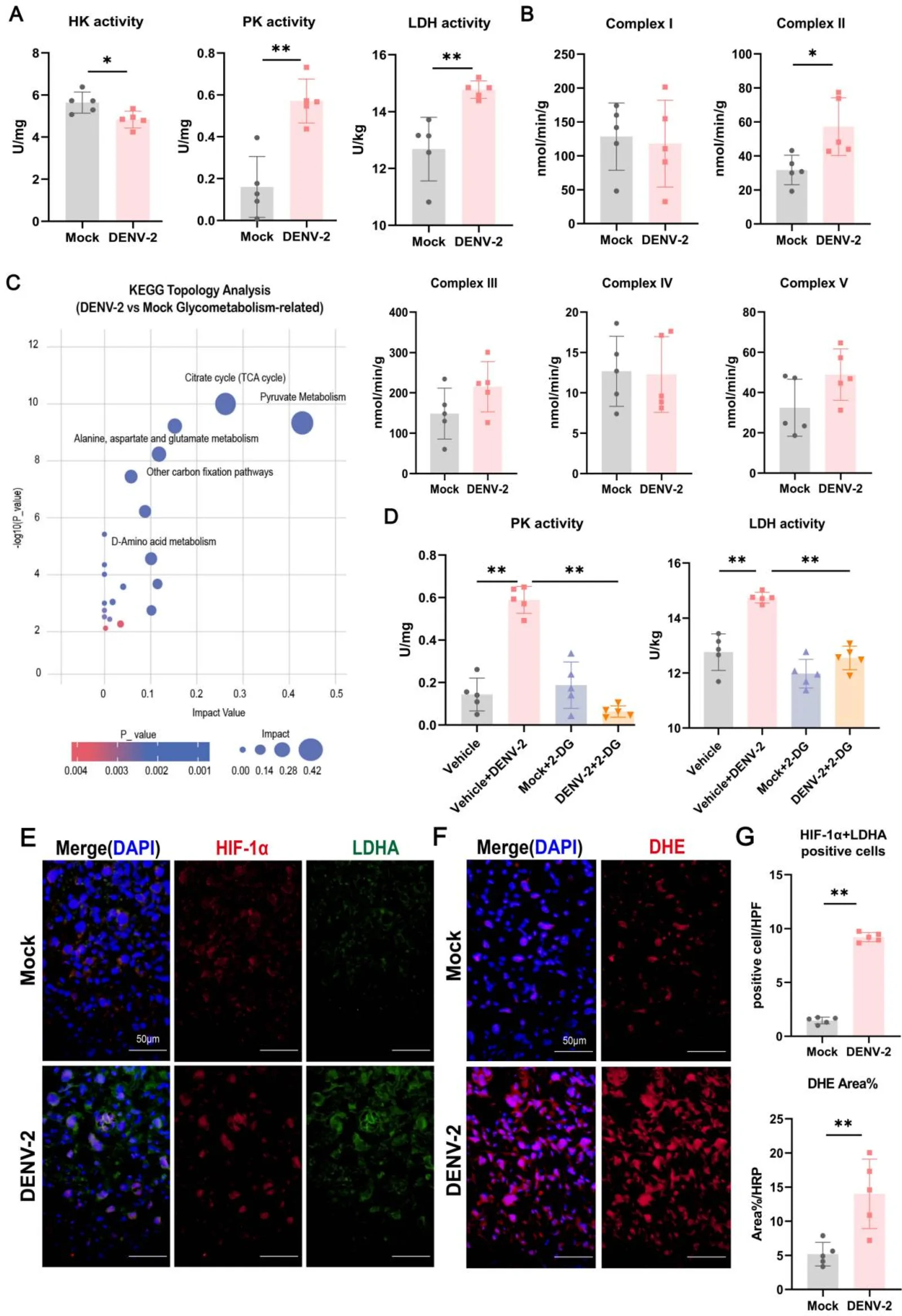
Underlying mechanism of the shift in respiratory mode in the placenta of DENV-2-infected *Ifnar*1^−/−^ pregnant mice. (**A**) Measurement of hexokinase (HK), pyruvate kinase (PK), and lactate dehydrogenase (LDH) activities in placental tissues of *Ifnar*1^−/−^ pregnant mice. (**B**) Assessment of Complex I–V activity in placental tissues of *Ifnar*1^−/−^ pregnant mice. (**C**) Bubble plot of KEGG Topology Analysis showing differential enrichment of gluconeogenesis-related metabolites between DENV-2-infected and Mock groups in placental metabolomics of *Ifnar*1^−/−^ pregnant mice. (**D**) PK and LDH activity in placental tissues of *Ifnar*1^−/−^ pregnant mice after 2-DG treatment. (**E**) Immunofluorescence analysis of Warburg effect activation in placental trophoblasts. Tissues were stained with anti-HIF-1α (red, marking hypoxic cells), anti-LDHA (green, a key Warburg effect enzyme), and DAPI (blue, nuclei). Scale bar, 50 μm. (**F**) Detection of ROS levels using the fluorescent probe DHE (red) with DAPI (blue) in placental tissues. Scale bar, 50 μm. (**G**) Quantification of LDHA^+^ cells and HIF-1α^+^ cells. Quantification of DHE area fractions. *N* = 5 per group. Data are presented as mean ± SD. Statistical significance was determined by a two-tailed unpaired Student’s *t*-test for two-group comparisons in panel D and by one-way ANOVA followed by Tukey’s multiple comparisons test for comparisons among multiple groups in panels A, B, and G. *P*-values: *<0.05, **<0.01.

Given that cellular respiration is a multistep process involving distinct enzymatic reactions and that key metabolic enzymes play pivotal roles in regulating the balance between oxidative phosphorylation and glycolysis (33), we next sought to define the enzymatic basis underlying the altered respiratory phenotype. Activities of major glycolytic enzymes (HK, PK, LDH) and OXPHOS complexes (I–V) were measured. PK and LDH activities were significantly increased in placentas from DENV-2-infected *Ifnar*1^−/−^ pregnant mice (Fig. 5A), confirming enhanced glycolytic flux and potentiation of the Warburg effect. In contrast, the activities of OXPHOS complexes I–V remained largely unchanged (Fig. 6B), indicating that mitochondrial structural integrity was preserved. However, under conditions of augmented glycolysis, mitochondria appeared functionally downweighed, suggesting a relative metabolic preference for glycolysis over OXPHOS. Complementary metabolomic profiling MetPA analyses further revealed the upregulation of pyruvate metabolism and the TCA cycle (Fig. 6C), suggesting an increased influx of glycolytic intermediates into mitochondrial metabolism. 2-DG treatment restored PK and LDH activities to near-normal levels (Fig. 6D) and enhanced the activities of mitochondrial Complexes I–V in placentas (Fig. S4A), confirming the causal role of glycolysis in this metabolic reprogramming. These results indicate that excessive glycolytic activation precedes and drives the metabolic shift from OXPHOS to the Warburg phenotype.

As LDHA is a key enzyme driving the Warburg effect (34–36), and HIF-1α has been identified as a major transcriptional regulator of its expression (37, 38), we next sought to identify the upstream factors responsible for LDHA activation. Immunofluorescence staining revealed increased expression of both HIF-1α and LDHA in placentas from DENV-2-infected mice (Fig. 6E through G). Since reactive oxygen species (ROS) are potent activators of HIF-1α (39, 40), superoxide levels were measured using the fluorescent probe dihydroethidium (DHE), revealing markedly elevated oxidative stress in DENV-2-infected placentas (Fig. 6F and G). To further assess the role of oxidative stress, an antioxidant mouse model was constructed by administering reduced glutathione (GSH) (Fig. S4B). Following antioxidant treatment, DHE fluorescence markedly decreased, accompanied by reduced HIF-1α and LDHA expression (Fig. S4C through E), indicating that ROS accumulation is a primary driver of HIF-1α activation and the downstream glycolytic reprogramming induced by DENV-2 infection.

To further assess the functional role of HIF-1α in trophoblast syncytialization, we performed *in vitro* experiments using BeWo cells. Pharmacological inhibition of HIF-1α with YC-1 significantly reduced HIF-1α and LDHA expression, accompanied by increased ATP levels. In addition, YC-1 treatment markedly enhanced trophoblast syncytialization, as indicated by elevated CD71 expression and β-hCG secretion. Consistently, the expression of fusion-related genes was upregulated (Fig. S5A through J). These results support a contributory role of HIF-1α in mediating oxidative stress-induced impairment of trophoblast fusion.

**Fig 7:**
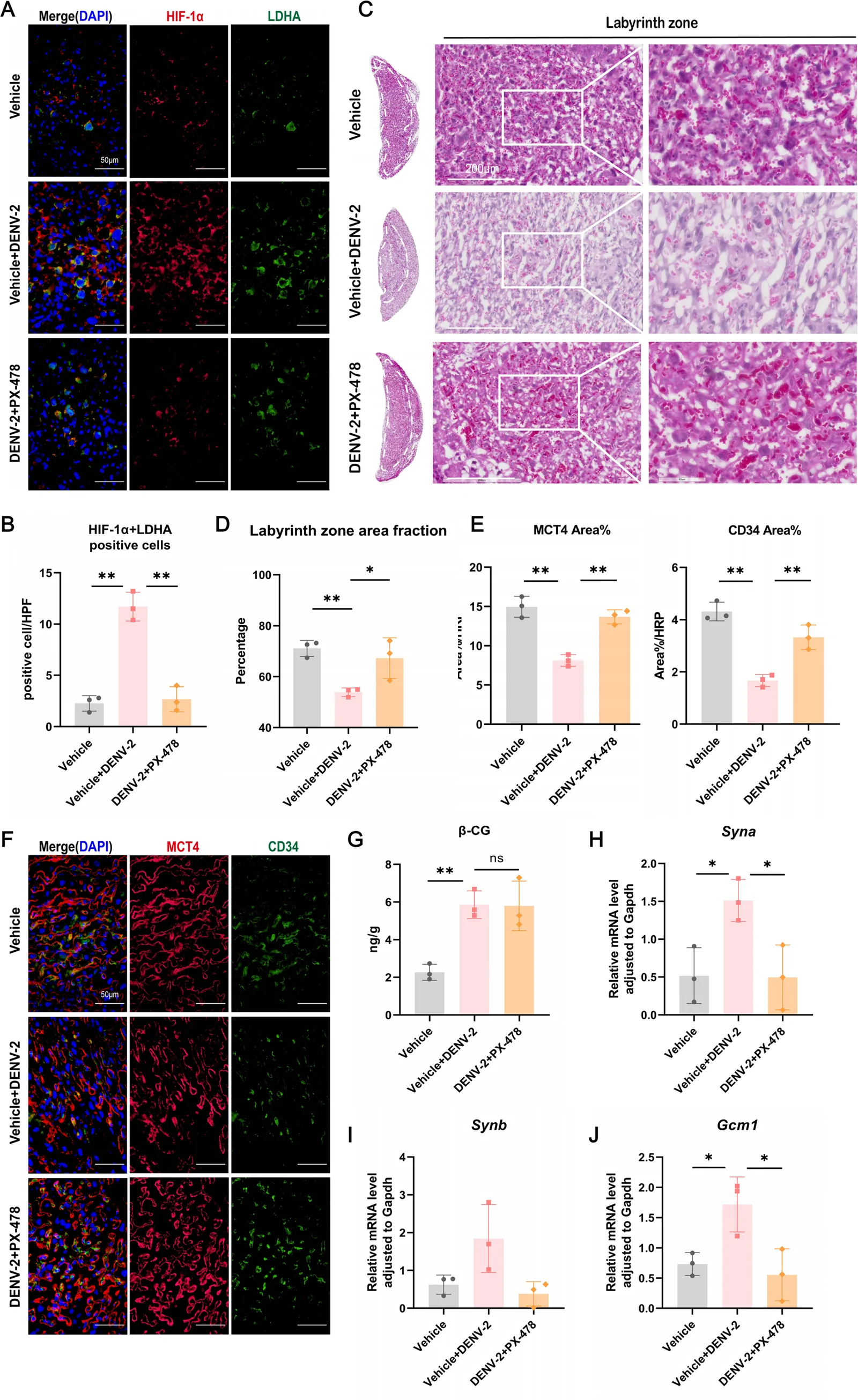
Pharmacological inhibition of HIF-1α rescues DENV-2-induced placental labyrinth zone injury and syncytialization defects in *Ifnar*1^−/−^ pregnant mice. (**A and B**) Representative immunofluorescence images and quantitative analysis of Warburg effect activation in placental trophoblasts after PX-478 treatment. Tissues were stained with anti-Hif1α (red, marking hypoxic cells), anti-LDHA (green, a key Warburg effect enzyme), and DAPI (blue, nuclei). Scale bar, 50 μm. (**C and D**) Representative H&E staining of PX-478-treated placental sections and quantitative analysis of the labyrinth zone area fraction across the indicated experimental groups. Scale bar, 200 μm. (**E and F**) Representative immunofluorescence staining and area percentage quantification of placental barrier and transport-related markers MCT4 (red) and CD34 (green). Scale bar, 50 μm. (**G**) ELISA quantification of β-CG levels in placentas after PX-478 treatment. (**H–J**) RT-qPCR analysis of fusogenic genes (*Syna, Synb, Gcm1*) in placentas from PX-478-treated *Ifnar*1^−/−^ mice. *N* = 3 per group. Data are presented as mean ± SD. Statistical analysis was performed using a two-tailed unpaired Student’s *t*-test. *P*-values: *<0.05, **<0.01. ns, not significant.

To ultimately establish the critical causal role of the HIF-1α-LDHA axis in DENV-2-induced placental dysfunction and syncytialization defects within a genuine viral infection context, we performed *in vivo* rescue experiments by introducing the HIF-1α-specific inhibitor, PX-478, into the DENV-2-infected mouse model. Immunofluorescence staining revealed that PX-478 treatment successfully abrogated the aberrant upregulation of HIF-1α and LDHA in placental tissues induced by DENV-2 (Fig. 7A and B). Crucially, the specific blockade of HIF-1α significantly rescued the histological damage in the placenta, as evidenced by a marked reversal and recovery of the pathological atrophy and reduced area percentage of the placental labyrinth zone caused by DENV-2 infection (Fig. 7C and D). Furthermore, PX-478 treatment effectively counteracted the loss of expression of the critical barrier and transporter proteins, MCT4 and CD34 (Fig. 7E and F), indicating robust protection of placental transport functions and vascular architecture. At the molecular and transcriptomic levels, the *in vivo* rescue experiment consistently demonstrated standardized regulatory effects (Fig. 7G through J); specifically, the transcriptional and secretory dysregulation of core placental syncytialization markers (the protein level of β-CG and the mRNA levels of *Syna*, *Synb*, and *Gcm1*) triggered by DENV-2 infection were significantly rectified upon PX-478 administration, returning nearly to the homeostatic levels of the mock control group. This compelling *in vivo* rescue evidence highly aligns with our *in vitro* cellular findings, directly establishing HIF-1α as a pivotal pathological mediator that drives DENV-2-induced placental barrier impairment and syncytialization abnormalities *in vivo*.

In summary, our findings reveal that DENV-2 infection induces ROS accumulation, which activates the HIF-1α-LDHA axis and drives a metabolic shift from oxidative phosphorylation to glycolysis. This ROS-mediated glycolytic reprogramming underlies placental metabolic dysfunction and may represent a critical mechanism linking DENV-2 infection to adverse pregnancy outcomes.

## DISCUSSION

Over the past 15 years, accumulating epidemiological and experimental evidence has linked DENV infection during pregnancy with adverse outcomes, including preterm delivery, fetal growth restriction, and low birth weight (41). Consistent with these observations, our study demonstrated that DENV-2 infection in pregnant *Ifnar*1^−/−^ mice resulted in IUGR without detectable viral replication in the placenta, suggesting an indirect pathogenic mechanism. Pregnant individuals infected with DENV remain at risk of obstetric complications, yet no vaccines or antiviral therapies are currently approved for use during pregnancy (42). Therefore, elucidating the mechanisms by which DENV impairs placental function is critical for developing targeted therapeutic strategies.

Adverse pregnancy outcomes arise from multiple mechanisms, including placental dysfunction (43), maternal vascular maladaptation (44), immune dysregulation (45), oxidative stress (46), and infection (47). However, mechanistic studies specifically linking DENV infection to adverse pregnancy outcomes remain relatively limited. In contrast to diverse pathogens, including Zika virus (48), SARS-CoV-2 (49), and Toxoplasma (50), which have all been reported to directly infect and damage trophoblasts, we observed limited DENV-2 distribution within the placental labyrinth zone. Instead, the absence of detectable viral replication in the placental labyrinth zone suggests that placental dysfunction may be mediated by host-derived factors following DENV-2 infection rather than direct viral invasion.

Morphological analyses revealed that DENV-2 infection leads to the marked disruption of trophoblast architecture in the placenta, accompanied by a significant reduction in syncytiotrophoblast area. Single-cell transcriptomic analysis further demonstrated that, although an increase in proliferating trophoblast progenitor cells was observed and the expression of syncytialization-associated markers, including *Syna*, *Synb*, *Gcm1*, and β-hCG, showed an overall upward trend, effective compensatory syncytialization was not achieved to restore the damaged trophoblast layer. Within the placental structure, trophoblast cells play a central role in maintaining maternal–fetal exchange and placental barrier function. Therefore, this study focused on the syncytiotrophoblast as the key functional unit of the placenta and proposes that impaired trophoblast syncytialization represents an important intermediate mechanism underlying DENV-2-induced IUGR.

The syncytiotrophoblast is continuously formed through the fusion of trophoblast progenitor cells, and its structural integrity and functional capacity are essential for maintaining placental exchange efficiency. Our results showed that DENV-2 infection did not significantly suppress the transcription of key syncytialization-associated genes, suggesting that its inhibitory effect on trophoblast syncytialization is unlikely to occur at the level of transcriptional regulation. Instead, DENV-2 may interfere with critical functional steps during the differentiation process, thereby disrupting the transition of trophoblast cells from a proliferative state to a fusion-competent state. These findings suggest that DENV-2 may indirectly impair trophoblast syncytialization by disrupting key intracellular physiological processes although the precise mechanisms remain to be further elucidated.

Our transcriptomic and metabolomic analyses revealed that DENV-2 infection reprograms placental energy metabolism toward a Warburg-like glycolytic phenotype. This shift was characterized by increased LDH activity, decreased ATP content, and elevated lactate accumulation, indicating a reliance on glycolysis despite preserved mitochondrial structure. In single-cell analysis, proliferating labyrinth trophoblast progenitors displayed upregulated glycolytic gene expression but failed to progress toward syncytial differentiation, suggesting that DENV-2 induces a glycolytic shift in placental trophoblasts and contributes to trophoblast dysfunction. In Zika virus infection, previous studies have demonstrated that the virus can directly impair trophoblast syncytialization (48) and induce metabolic rewiring in trophoblasts, including increased glucose utilization (51), suggesting a potential mechanistic link between virus-induced metabolic reprogramming and defective syncytialization. In contrast, under conditions of limited DENV infection within the placental labyrinth zone, the observed glycolytic alterations are more likely to be driven by host-derived factors rather than direct viral effects on trophoblasts.

To investigate the role of glycolysis in this pathological process, we applied 2-deoxy-D-glucose (2-DG) in a pregnant mouse model of DENV-2 infection to functionally assess the effects of glycolytic inhibition on trophoblast function and placental metabolism. 2-DG is a commonly used inhibitor of glycolysis. Following 2-DG treatment, placental lactate levels were significantly reduced, while ATP levels were significantly increased. In parallel, syncytiotrophoblast density was improved, and partial restoration of the labyrinth zone architecture was observed. Although 2-DG and its derivatives have been studied as metabolic modulators in cancer therapy due to the widespread presence of the Warburg phenotype in tumor cells (52), their application in infectious diseases remains relatively limited. A few studies have suggested that glycolysis may influence viral replication or host inflammatory responses in certain viral infection models (53, 54); however, its role in DENV-associated tissue injury, particularly in pregnancy-related complications, has not been systematically investigated. Therefore, our findings suggest that glycolytic metabolism may represent a potential modulatory pathway in DENV-associated placental dysfunction.

Previous studies have shown that under conditions of cellular stress or injury, cells preferentially rely on glycolysis to rapidly generate energy and metabolic intermediates, accompanied by a relative reduction in mitochondrial oxidative phosphorylation (52). In the present study, we observed a significant increase in oxidative stress levels in placental tissues following DENV-2 infection. Oxidative stress is recognized as a key pathological feature of DENV infection. Clinical studies have also reported elevated levels of reactive oxygen species (ROS) in the plasma and peripheral blood cells of patients with DENV infection, which correlate with disease severity (55). During pregnancy, the placenta is highly sensitive to redox homeostasis. While moderate levels of ROS play a regulatory role in placental development, excessive accumulation of ROS can disrupt placental homeostasis. Previous studies have demonstrated that elevated ROS levels can inhibit trophoblast proliferation and differentiation, induce apoptosis, and impair placental endothelial function, ultimately affecting placental perfusion and leading to adverse pregnancy outcomes, including preterm birth and IUGR (56, 57).

ROS, as key signaling molecules in metabolic reprogramming, can stabilize hypoxia-inducible factor 1α (HIF-1α) and promote its accumulation in the nucleus (58). As a central transcriptional regulator of glucose metabolism, HIF-1α directly activates the transcription of multiple glycolysis-related genes, including glucose transporter 1 (GLUT1), hexokinase 2 (HK2), and lactate dehydrogenase A (LDHA) (59, 60). Among these, LDHA catalyzes the terminal step of glycolysis and plays a critical role in sustaining high glycolytic flux. Our results showed that the expression levels of HIF-1α and LDHA were increased in placental tissues following DENV-2 infection. Furthermore, *in vivo* antioxidant intervention using glutathione (GSH) to suppress ROS, as well as pharmacological inhibition of HIF-1α using YC-1 in BeWo cells, both reduced the expression of HIF-1α and LDHA. These findings support a role for the ROS-HIF-1α-LDHA signaling axis in promoting glycolytic activation in trophoblasts following DENV-2 infection, thereby contributing to placental dysfunction.

Similar mechanisms have been reported in other viral infection models. For example, in influenza virus infection, enhanced glycolysis and upregulation of HIF-1α and LDHA have been observed in A549 epithelial cells (53), while in Zika virus infection, oxidative stress-related pathways (e.g., Nrf2) are involved in regulating viral replication in A172 human astrocytoma cells (61). Collectively, these studies indicate that ROS-mediated stabilization of HIF-1α can promote LDHA expression and lactate production, driving a Warburg-like metabolic state. Notably, in contrast to viruses that directly infect target cells, DENV-2 appears to induce similar metabolic alterations under conditions of limited placental infection, suggesting a distinct, host-mediated mechanism. This finding provides insight into a potential mechanism underlying adverse pregnancy outcomes associated with DENV infection.

This study systematically elucidates that, following DENV-2 infection, the ROS-HIF-1α-LDHA signaling axis contributes to placental dysfunction by promoting glycolytic reprogramming in trophoblasts and impairing syncytialization. However, several limitations should be acknowledged. (i) Wild-type mice are naturally resistant to DENV infection; therefore, the *Ifnar*1^−/−^ mouse model, which lacks type I interferon signaling, remains a necessary and widely used system for studying DENV pathogenesis. However, the absence of type I interferon signaling markedly compromises host antiviral responses, leading to enhanced viral replication and heightened host stress responses, which may amplify infection-associated pathological phenotypes (62). As a result, the observed effects may differ from those occurring in human pregnancy. (ii) The placenta exhibits substantial differences in structure, metabolism, and immune characteristics across gestational stages. Clinical evidence indicates that DENV infection during early pregnancy is more frequently associated with miscarriage, whereas infection during mid-to-late pregnancy is more commonly linked to fetal growth restriction and placental dysfunction, suggesting that distinct stages of placental development may correspond to different pathogenic mechanisms and phenotypes (41, 63). In this study, we focused on late gestation when the placental labyrinth is functionally established, thereby minimizing confounding effects related to ongoing placental development. In addition, previous studies in early pregnancy mouse models have shown that antibody-dependent enhancement (ADE) can increase the risk of fetal infection and death (64). Future studies are warranted to define stage-specific mechanisms of DENV-induced placental dysfunction. (iii) In this study, 2-DG and GSH were used as functional modulators to interrogate the underlying mechanisms. Although both agents showed encouraging effects in modulating the observed phenotypes, they are associated with potential toxicity, and their clinical application should be interpreted with caution.

In summary, this study demonstrates that DENV-2 infection induces oxidative stress in the placenta, which drives a metabolic shift in cellular respiration from oxidative phosphorylation to the Warburg effect. This glycolytic reprogramming limits ATP production required for trophoblast syncytialization, impairs syncytiotrophoblast-mediated nutrient and gas exchange, and consequently results in intrauterine growth restriction. This work reveals a novel mechanism by which DENV infection leads to adverse pregnancy outcomes, highlighting potential strategies for clinical intervention. In addition, it offers a new perspective for exploring the pathogenic pathways of other non-vertically transmitted infectious agents that can adversely affect pregnancy.

## Data Availability

The scRNA-seq data generated in this study have been deposited in the National Microbiome Data Center and are publicly available under accession number NMDC40077531.

## SUPPLEMENTAL MATERIAL

Fig. S1 jvi.00300-26-s0001.tif Supplementary analyses of DENV-2 infection in *Ifnar*1^-/-^ pregnant mice.

Fig. S2 jvi.00300-26-s0002.tif Characterization of viral distribution, single-cell landscape, and trophoblast transcriptomic alterations in DENV-2-infected placentas.

Fig. S3 jvi.00300-26-s0003.tif Metabolic alterations in *Ifnar*1^-/-^ placentas following DENV-2 infection and their improvement by 2-DG treatment.

Fig. S4 jvi.00300-26-s0004.tif Changes in respiratory enzymes and intermediate metabolites in the placentas of *Ifnar*1^-/-^ pregnant mice.

Fig. S5 jvi.00300-26-s0005.tif Pharmacological inhibition of HIF-1α partially rescues oxidative stress-induced metabolic reprogramming and syncytialization defects in BeWo cells.

Supplemental legends jvi.00300-26-s0006.docx Legends for Fig. S1 to S5.

Table S1 jvi.00300-26-s0007.docx Primer sequences used for RT-qPCR analysis.

ASM does not own the copyrights to Supplemental Material that may be linked to, or accessed through, an article. The authors have granted ASM a non-exclusive, world-wide license to publish the Supplemental Material files. Please contact the corresponding author directly for reuse.
