# Supplementary material for: Trophoblast metabolic reprogramming triggers adverse pregnancy outcomes during DENV-2 infection in mice
Source: J Virol. 2026 Aug 21;100(9):e00300-26. doi: 10.1128/jvi.00300-26 (PMC13595951; doi:10.1128/jvi.00300-26)
Supplement: Supplementary file 1 [file jvi.00300-26-s0006.docx]

**Supplementary Figure Legend**

**Supplementary Figure S1. Supplementary analyses of DENV-2 infection in *Ifnar*1^-/-^ pregnant mice**

(A) Schematic of the infection model. *Ifnar*1^-/-^ pregnant mice were inoculated by footpad injection with 10^5^ PFU of DENV-2 at embryonic day (E)12.5; mock group received PBS. Maternal blood and placentas were collected at E15.5 and E18.5.

(B) Maternal body weight changes from E0.5 to E18.5.

(C) Gross morphology of fetuses . Left: representative images of embryos and placentas at E18.5, with crown-rump length indicated by the white line. Right: quantification of crown-rump length.

(D) ELISA measurement of VEGF and PGF levels in placentas.

N = 5 per group. Data are presented as mean ± SD. Statistical analysis was performed using a two-tailed unpaired Student’s T-test. P-values: **<0.01.

**Supplementary Figure S2. Characterization of viral distribution, single-cell landscape, and trophoblast transcriptomic alterations in DENV-2-infected placentas**

(A) Quantification of viral RNA levels in maternal blood and placental tissue by RT-qPCR. N=4 per group.

(B) UMAP visualization of single-cell RNA-seq data from placental tissues, annotated by major cell types. Feature plot showing the distribution of DENV-2-derived transcripts across cell populations.Viral signals are detected in a small subset of cells and are predominantly enriched in macrophages (red circle).

(C) Regional analysis of placental compartments showing minimal 4G2 signal in the labyrinth layer and relatively higher signal in the decidual region in DENV-2-infected group. Scale bar, 50 μm.

(D) UMAP plot showing *Krt8*^+^ cell populations across the placenta. Upper: Clusters in the lower right and central regions exhibited high expression; *Krt8*^+^ cells were extracted as trophoblasts. Lower: UMAP visualization of trophoblasts after subclustering. Total trophoblast number was reduced in DENV-2-infected placentas.

(E) Violin plots comparing expression of apoptosis-, ferroptosis-, and autophagy-related genes. Heatmap of top marker genes for each subpopulation.

(F) Heatmap of top marker genes across trophoblast subpopulations.

**Supplementary Figure S3. Metabolic alterations in *Ifnar*1^-/-^ placentas following DENV-2 infection and their improvement by 2-DG treatment**

(A) GSEA-KEGG analysis of differentially expressed genes in LaTPs from DENV-2–infected versus mock groups.

(B) Dotplot showing expression of OXPHOS and Warburg pathway-related genes in LaTPs from DENV-2-infected and mock groups.

(C) Principal component analysis (PCA) of placental metabolomics data. Each point represents a sample, with red indicating DENV2-infected samples and blue indicating mock samples. The ellipses represent 95% confidence intervals for each group. PC1 and PC2 explain 47.30% and 15.20% of the total variance, respectively.

(D) ELISA quantification of angiogenic factors VEGF and PlGF in placentas from *Ifnar*1^-/-^ mice after 2-DG treatment.

N = 5 per group. Data are presented as mean ± SD. Statistical significance was determined by one-way ANOVA followed by Tukey’s multiple comparisons test. P-values: **<0.01.

**Supplementary Figure S4. Changes in respiratory enzymes and intermediate metabolites in the placentas of *Ifnar*1^-/-^ pregnant mice**

(A) Activities of mitochondrial complexes I-V in placental tissues of *Ifnar*1^-/-^ pregnant mice after 2-DG treatment.

(B) Experimental design: pregnant *Ifnar*1^-/-^ mice were injected via the footpad with 10^5^ PFU DENV-2 or PBS on embryonic day 12.5 (E12.5). From the day of infection until E18.5, mice received daily intraperitoneal injections of GSH. Blood and placentas were collected at E18.5 for downstream analyses.

(C) Detection of ROS levels using the fluorescent probe DHE (red) with DAPI (blue) in placental tissues after GSH treatment. Scale bar, 50 μm.

(D) Immunofluorescence analysis of Warburg effect activation in placental trophoblasts after GSH treatment. Tissues were stained with anti-HIF-1α (red, marking hypoxic cells), anti-LDHA (green, a key Warburg effect enzyme), and DAPI (blue, nuclei). Scale bar, 50 μm.

(E) Quantification of LDHA^+^ cells and HIF-1α^+^ cells in placental tissues of *Ifnar*1^-/-^ pregnant mice after GSH treatment. Quantification of DHE area fractions in placental tissues of *Ifnar*1^-/-^ pregnant mice after GSH treatment.

N = 5 per group. Data are presented as mean ± SD. Statistical significance was determined by one-way ANOVA followed by Tukey’s multiple comparisons test. P-values: **<0.01.

**Supplementary Figure S5.** **Pharmacological inhibition of HIF-1α partially rescues oxidative stress-induced metabolic reprogramming and syncytialization defects in BeWo cells**

(A) Representative immunofluorescence images showing HIF-1α (red) and LDHA (green) expression in BeWo cells treated with increasing concentrations of YC-1 (0, 5, 10, and 20 μM). Nuclei were stained with DAPI (blue). Yellow arrows indicate representative cells with high HIF-1α/LDHA signal. Scale bar, 100 μm.

(B) Quantification of HIF-1α and LDHA fluorescence intensity normalized to DAPI (HIF-1α and LDHA/DAPI ratio).

(C) Intracellular ATP levels measured in BeWo cells under different YC-1 treatments.

(D) Quantification of CD71 fluorescence intensity normalized to DAPI (CD71/DAPI ratio).

(E) Lactate levels in cell culture supernatants.

(F) Representative immunofluorescence images of CD71 (red) in BeWo cells treated with increasing concentrations of YC-1. Nuclei were stained with DAPI (blue). Scale bar, 100 μm.

(G) Secretion of human chorionic gonadotropin (β-hCG) in culture supernatants.

(H-J) Relative mRNA expression levels of fusion-related genes *ERVW-1*, *ERVFRD-1*, and *GCM1*, normalized to *Gapdh*.

N = 5 per group. Data are presented as mean ± SD. Statistical significance was determined by one-way ANOVA followed by Tukey’s multiple comparisons test for panels (B), (C), (D), (E), and (G), and by the Mann–Whitney U test for panels (H), (I), and (J). P-values: *<0.05, **<0.01.
