# Supplementary material for: Trophoblast metabolic reprogramming triggers adverse pregnancy outcomes during DENV-2 infection in mice
Source: J Virol. 2026 Aug 21;100(9):e00300-26. doi: 10.1128/jvi.00300-26 (PMC13595951; doi:10.1128/jvi.00300-26)
Supplement: Supplementary file 2 [file jvi.00300-26-s0007.docx]

**Supplementary Table**

**Supplementary Table S1**. Primer sequences used for RT-qPCR analysis.

F, forward primer; R, reverse primer.

| Gene | primer oligonucleotides (5’-3’) |
| --- | --- |
| DENV-2-F | CATGGGTAACTTATGGGAC |
| DENV-2-R | GTTTCAGTTCGTGTCTCCA |
| GAPDH-F | GCATTGTGGAAGGGCTCA |
| GAPDH-R | ACCAGTGGATGCAGGGAT |
| mGcm1-F | CCCCAGCAAGTTCCATCAGA |
| mGcm1-R | AAGGCTCACCTCCCGGATT |
| Syna-F | AGATACCCCGATGACCACGTC |
| Syna -R | TGAGGATCGTCTGGGTGGAG |
| Synb -F | CCACCACCCATACGTTCAAA |
| Synb -R | GGTTATAGCAGGTGCCGAAG |
| GCM1-F | ACCTGCCATCTGTGACAAGG |
| GCM1-R | GCCTTCCTGGAAAGACCAAG |
| ERVW-1-F | ACCCTCCGTACCCATACTCG |
| ERVW-1-R | GGGAGGAGTTACCCACCTGA |
| ERVFRD-1-F | CCATTTTCGGACCCATCTTT |
| ERVFRD-1-R | CCGCTGTAGAGCTGAGGTTG |
